# Nummular Eczema of Breast: A Potential Dermatologic Complication after Mastectomy and Subsequent Breast Reconstruction

**DOI:** 10.1155/2015/209458

**Published:** 2015-08-24

**Authors:** Yoshiko Iwahira, Tomohisa Nagasao, Yusuke Shimizu, Kumiko Kuwata, Yoshio Tanaka

**Affiliations:** ^1^Tokyo Breast Surgery Clinic, Japan; ^2^Department of Plastic and Reconstructive Surgery, Faculty of Medicine/Graduate School of Medicine, Kagawa University, Kida County, Miki-Cho, Ikenobe 1750-1, Takamatsu, Kagawa 761-0793, Japan; ^3^Department of Plastic and Reconstructive Surgery, Ryukyu University Hospital, Japan; ^4^Department of Plastic Surgery, Aichi Children's Health and Medical Center, Japan

## Abstract

*Purposes*. The present paper reports clinical cases where nummular eczema developed during the course of breast reconstruction by means of implantation and evaluates the occurrence patterns and ratios of this complication. *Methods*. 1662 patients undergoing breast reconstruction were reviewed. Patients who developed nummular eczema during the treatment were selected, and a survey was conducted on these patients regarding three items: (1) the stage of the treatment at which nummular eczema developed; (2) time required for the lesion to heal; (3) location of the lesion on the reconstructed breast(s). Furthermore, histopathological examination was conducted to elucidate the etiology of the lesion. *Results*. 48 patients (2.89%) developed nummular eczema. The timing of onset varied among these patients, with lesions developing after the placement of tissue expanders for 22 patients (45.8%); after the tissue expanders were replaced with silicone implants for 12 patients (25%); and after nipple-areola complex reconstruction for 14 patients (29.2%). Nummular eczema developed both in periwound regions (20 cases: 41.7%) and in nonperiwound regions (32 cases: 66.7%). Histopathological examination showed epidermal acanthosis, psoriasiform patterns, and reduction of sebaceous glands. *Conclusions*. Surgeons should recognize that nummular eczema is a potential complication of breast reconstruction with tissue expanders and silicone implants.

## 1. Introduction

Breast cancer is one of the most common cancers. For instance, in the United States, one out of eight females develops breast cancer in her life [1]. Total mastectomy is conducted for patients in whom the tumor extends to a large portion of the mammary gland. Since the breast symbolizes femininity, its loss can inflict serious psychological damage on patients. For this reason, reconstruction of the lost breast is generally performed. Methods of breast reconstruction are classified into two genres: reconstruction by the transfer of autologous tissues [2, 3] and reconstruction with artificial materials [4–6]. The latter genre is less invasive than the former. However, breast reconstruction with artificial materials is sometimes accompanied by postoperative complications. Well-known complications include exposure of expanders or silicone implants. Besides these complications, we have recognized that nummular eczema may develop on the skin of the reconstructed breast, although this is not widely noted. Nummular eczema is a skin lesion first reported by Marie Guillaume Alphonse Devergie in the 19th century [7–9]. The present paper aims to introduce clinical cases of nummular eczema that developed during the breast reconstruction process, and it discusses how we can prevent this complication.

## 2. Methods

A total of 1,662 patients operated on for breast reconstruction at Tokyo Breast Surgery Clinic between February 2008 and October 2013 were surveyed. All these patients were Japanese females. Among these 1,662 patients, 48 patients developed lesions diagnosed by dermatologists as nummular eczema on the skin of the reconstructed breasts. These 48 patients were reviewed in the present study. This study was approved by the ethical committee of the institutional review board of Tokyo Breast Surgery Clinic, and written informed consent was obtained from all patients. Evaluation was performed regarding the following issues.

### 2.1. Onset

Breast reconstruction is performed in three steps. In the first step, tissue expanders are placed in the layer beneath the major pectoral muscle to expand the skin; in the second step, the expanders are replaced with silicone implants; in the third step, the nipple-areola complex is reconstructed using skin graft and local flaps. Based on at which of these three steps nummular eczema developed, the 48 patients were classified into the following three groups.

*Group 1.* This group included patients who developed nummular eczema after the placement of tissue expanders and before replacement with silicone implants.

*Group 2.* This group included patients who developed nummular eczema after implant placement and before reconstruction of the nipple-areola complex.

*Group 3.* This group included patients who developed nummular eczema after reconstruction of the nipple-areola complex.

The intervals between the operation preceding development and development of nummular eczema were evaluated for each of these groups.

### 2.2. Comorbidities and Medications

Comorbidities, medications, and absence/presence of atopic dermatitis were evaluated for each of the three groups.

### 2.3. Location of Lesion

Placement of the tissue expander and its subsequent replacement with an implant are conducted through the wound made in the initial mastectomy. The skin regions of the breast were divided into two regions: the region adjacent to the wound and the region away from the wound. The former region was termed the periwound region; the latter region was termed the nonperiwound region. For patients belonging to each of the three groups, the locations of lesions were evaluated based on this classification. For patients belonging to Group 3, the nonperiwound region was further subdivided into the nipple-areola complex and the other part, and the location of nummular eczema was evaluated.

### 2.4. Healing Time

All patients were treated with a vaseline-based ointment containing 0.12% betamethasone valerate. For the patients belonging to each of the three groups, the time needed to heal was evaluated.

### 2.5. Histopathological Evaluation

Harvesting skin pieces from the lesion to examine histological changes occurring in the lesion of the skin was suggested to the 48 patients who developed nummular eczema. Two patients elected to receive the histopathological examination, and samples of the lesions were harvested from them. These samples were examined by means of haematoxylin-eosin staining and azan staining.

## 3. Results

### 3.1. Onset

**Figure 1:**
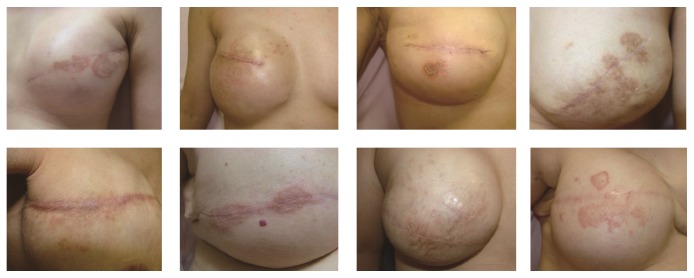
Representative cases of patients who developed nummular eczema after placement of tissue expanders and before replacement with silicone implants.

**Figure 2:**
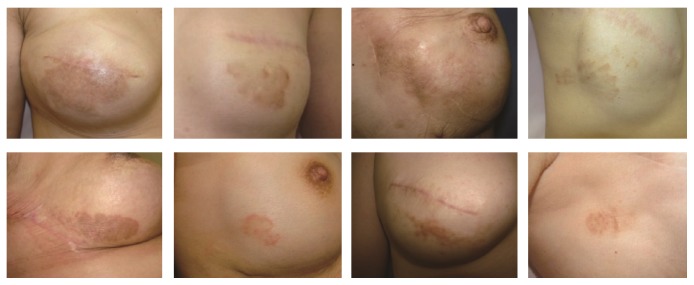
Representative cases of patients who developed nummular eczema after placement of silicone implants and before reconstruction of the nipple-areola complex.

**Figure 3:**
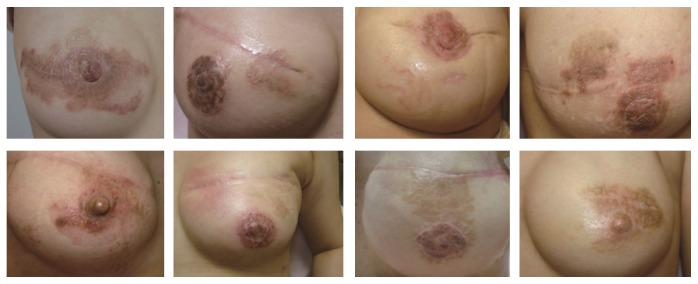
Representative cases of patients who developed nummular eczema after reconstruction of nipple-areola complex.

The onset timing presented variation, with 28 cases (45.8%) developing nummular eczema after placement of tissue expanders (Group 1), 12 cases (25.0%) developing lesions after replacement of expanders with silicone implants (Group 2), and 14 cases (29.2%) developing lesions after reconstruction of the nipple-areola complex (Group 3). Pictures of representative cases for each of the three groups are provided in Figures 1–3, respectively.

Intervals between the development of nummular eczema and the nearest preceding operation are listed in Table 1.

### 3.2. Medication of the Patients

#### 3.2.1. Comorbidities

Among the 48 patients who had nummular eczema, 47 patients had no comorbidities. One patient in Group 2 had rheumatoid arthritis. No patient had underlying dermatological disease, such as atopic dermatitis.

#### 3.2.2. Medications

18 patients were taking tamoxifen citrate; two patients were taking anastrozole. The distribution of these patients is shown in Table 2. The patient who had rheumatoid arthritis was taking prednisolone as treatment for the disease.

### 3.3. Location

Nummular eczema developed for both periwound regions (20 cases: 41.7%) and nonperiwound regions (32 cases: 66.7%). In four cases, the lesion extended to both regions. Distribution of the locations for each group is demonstrated in Table 3.

### 3.4. Healing Time

The average and standard deviation of the time the affected skin took to heal are presented in Table 1. In a majority of cases, nummular eczema healed within three to four weeks with the administration of steroid ointments (betamethasone valerate). In no cases did nummular eczema further deteriorate to cause ulceration or chronic infection after application of steroid ointment.

### 3.5. Histopathological Findings

**Figure 4:**
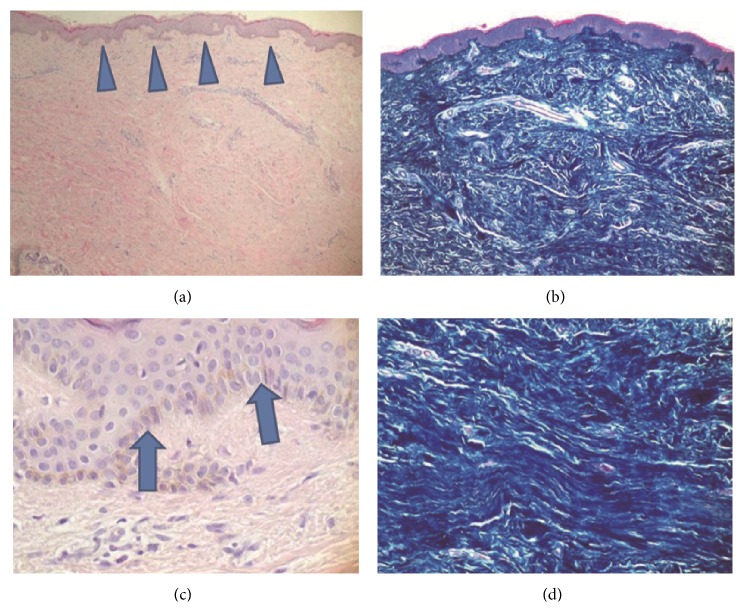
Histological findings of nummular eczema on the breast skin. (a) H-E staining magnified by 40 times. Epidermal acanthosis is shown (triangular arrows). (c) H-E staining magnified by 400 times. A psoriasiform pattern with hyperkeratosis (arrows), hypergranulosis, and minimal parakeratosis is observed. (b) Azan staining magnified by 40 times. Reduction of sebaceous glands is noted. (d) Azan staining magnified by 100 times. Randomly aligned hypertrophy of collagen fibers is observed.

Hematoxylin and eosin staining (Figure 4, left column) reveals significant epidermal acanthosis. Furthermore, psoriasiform patterns with hyperkeratosis, hypergranulosis, and minimal parakeratosis were also observed (Figure 4, left and right columns). Azan staining showed unidirectionally aligned collagen fibers, which formed the fibrosis.

## 4. Discussion

Nummular eczema is a discoid lesion of the skin that most frequently develops in the upper and lower extremities, as well as on the trunk, dorsum of the hand, face, and neck [10]. The etiological cause of nummular eczema remains unclear, and it is recognized as a multifactorial clinicomorphological entity, not an independent disease* per se* [8, 9]. Nummular eczema often takes a chronic and recursive course. Various causes such as atopic dermatitis, dry skin, mental stress, the weather, infection, and alcohol can induce nummular eczema [11–14].

Herein, the etiological causes of the nummular eczema in our series are discussed. Nummular eczema rarely occurs in the breast region, and it only develops there in limited situations, according to our review of the existing literature. Specifically, side effects caused by combined usage of interferon alfa-2b and ribavirin [15, 16], extramammary Paget's disease [17], and hematological diseases [18] are reported to have caused nummular eczema in the breast region. No patient in our series had any of these background factors. Among the 48 nummular eczema patients, 18 patients took tamoxifen citrate, and two patients took anastrozole as supplemental medication for breast cancer. Review of the literature revealed no evidence of relationship between the intake of these drugs and the development of nummular eczema.

When foreign materials are placed in the human body, this induces an immunological response and provokes disorders, including skin lesions. This condition is called human adjuvant disease [19]. Since silicone implants are foreign to the human body, it is possible that the nummular eczema in our cases was a symptom of human adjuvant disease, which causes abnormal granulation to develop at sites where foreign material contacts the body. However, none of our patients developed such granulation. With human adjuvant disease, skin lesions do not heal as long as the foreign material remains in the human body. However, all our patients healed with application of steroid ointments, without removing the silicone implants. Hence, it seems unlikely that nummular eczema in our cases was caused by immunological response.

**Figure 5:**
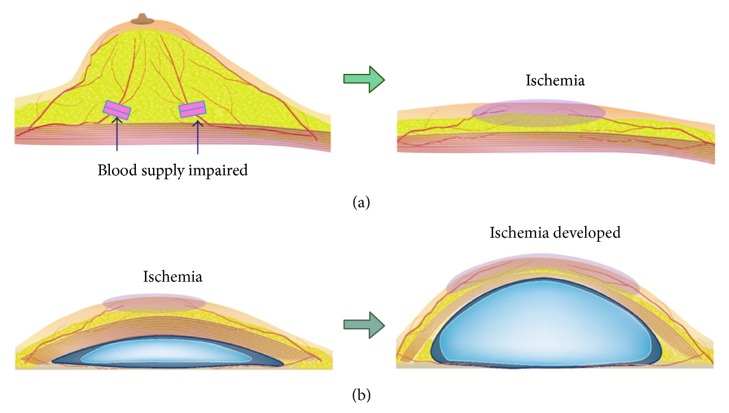
(a) Mastectomy impairs blood supply from the thoracic wall to the breast skin. (b) Embedment of an expander and expansion induces transient ischemia of the breast skin.

We speculate that nummular eczema was induced not by the intrinsic factors discussed above, but by structural change of the skin due to previous surgeries. When mastectomy is conducted, the skin of the breast region is impaired by two mechanisms. First, operative maneuvers damage the skin. Mastectomy is performed through a short incision for cosmetic reasons. When surgical approach is conducted through a short incision, the incision margin is strongly compressed with retractors to expose the operative field, damaging the skin at the margin of the incision. Second, the blood supply to the breast skin is reduced as a result of mastectomy. Usually, blood supply to the breast skin is provided through two routes. With the first route, the blood supply comes from regions surrounding the breast through vessels of the subdermal fat layer; with the second route, the blood supply comes from the thoracic wall through the mammary gland (Figure 5, top left). The second pathway is impaired by the removal of the mammary gland. Accordingly, ischemia occurs on the breast skin (Figure 5, top right).

Previously, we have conducted histopathological research regarding the effects on the breast skin of radiation for the treatment of breast cancer [20]. This study revealed that radiation induces hyperplasia of the epidermis, flattening of the papillary layer, atrophy of the dermal appendages, high density of dermal collagen fibers, and unidirectional alignment of dermal collagen fibers. On the other hand, the study also demonstrated that atrophy of dermal appendages, high density of dermal collagen fibers, and unidirectional alignment of dermal collagen fibers can also develop with the breast skin even when radiation is not used. Namely, whether radiated or not, skin of the breast region is damaged after mastectomy. It is speculated that the damage is caused by the reduction of blood supply induced by the two mechanisms described above.

Besides damage by the mastectomy, temporary ischemia due to subsequent expansion of the skin may be a causative factor for nummular eczema. Ischemia deteriorates the function of the sebaceous glands, making it difficult for the skin to retain its moisture. Hence, the skin becomes susceptible to mechanical stimulation caused by friction with underwear or clothes, increasing the risk of developing nummular eczema. Viewed this way, it is hypothesized that the thickness of the subdermal fat can also be an influential factor for the development of nummular eczema. In patients whose subdermal fat layers are thin, the blood supply to the breast skin may be significantly reduced as expansion proceeds. On the other hand, in patients who have thick fat layers, the blood supply to the skin is less likely to be reduced by skin expansion, because the thick subdermal layer functions as a shock absorber. The patients included in the present study were all Japanese females. Since fat layers are thinner in Asian patients than in Caucasian patients, it is possible that the occurrence ratio of nummular eczema is higher in the present study than it would be for other ethnicities. It is desirable that the influence of the patients' ethnicity be elucidated in future studies.

With all patients in our series, nummular eczema was successfully cured with the application of steroid ointments. Infection of the embedded materials and skin necrosis are well known as major complications of breast reconstruction with tissue expanders and silicone implants. Nummular eczema might appear minor compared to these complications. However, patients with breast cancer are often nervous because of their uneasiness about the disease [21, 22]. Furthermore, an extended period—as long as six months—may be required for nummular eczema to heal in some patients (Table 2). Hence, even though nummular eczema does not directly lead to failure of the treatment, occurrence of this complication can negatively affect the relationship between the patient and physician. Breast surgeons and plastic surgeons should recognize nummular eczema as a potential complication of breast reconstruction—which we name Iwahira's eczema—and take measures to prevent its occurrence. For the prevention of nummular eczema, constant skin care with regular cleansing and moisturizers is recommended [23].

## 5. Conclusion

Nummular eczema can develop during breast reconstruction using tissue expanders and silicone implants. It is speculated that this dermatologic complication is induced by the deterioration of the sebaceous glands' functioning due to the change of the skin structure caused by surgical intervention. Breast surgeons and plastic surgeons should recognize nummular eczema as a potential complication of breast reconstruction using artificial materials.

## Figures and Tables

**Table 1:** Ages, onset timing, and healing times. The figures in each column indicate averages and standard deviations. Onset timing means the interval between the development of nummular eczema and the last operation before onset.

	Age	Onset timing (weeks)	Healing time (weeks)
Group 1 (*n* = 22)	45.2 ± 9.0 SD	32.1 ± 21.5 SD	3.5 ± 1.4 SD
Group 2 (*n* = 12)	41.1 ± 7.7 SD	84.2 ± 107.4 SD	4.4 ± 2.6 SD
Group 3 (*n* = 14)	46.7 ± 11.5 SD	64.4 ± 61.9 SD	3.2 ± 2.8 SD

**Table 2:** Medication.

	Tamoxifen citrate	Anastrozole
Group 1 (*n* = 22)	13	2
Group 2 (*n* = 12)	3	0
Group 3 (*n* = 14)	2	0

**Table 3:** Location of lesions (since some patients had nummular eczema in more than one region, the sums of the columns do not necessarily match the patient's numbers for each group).

	Periwound region	Nonperiwound region	
Group 1 (*n* = 22)	9	15	
Group 2 (*n* = 12)	4	10	
Group 3 (*n* = 14)	7	Reconstructed nipple-areolar complex	7
		Non-NAC region	7
